# Value of the Barthel scale in prognostic prediction for patients with cerebral infarction

**DOI:** 10.1186/s12872-019-01306-1

**Published:** 2020-01-13

**Authors:** Qun-Xi Li, Xiao-Jing Zhao, Yan Wang, Da-Li Wang, Jiang Zhang, Tie-Jun Liu, Yan-Bo Peng, Hai-Yan Fan, Fu-Xia Zheng

**Affiliations:** 1 Department of Neurosurgery, Affiliated Hospital of North China University of Science and Technology, Tangshan, 063000 China; 2 Department of Neurology, Affiliated Hospital of North China University of Science and Technology, No. 73 of Jianshe South Road, Lubei District, Tangshan, 063000 China; 3 Department of Anesthesia, Affiliated Hospital of North China University of Science and Technology, Tangshan, 063000 China

**Keywords:** Stroke, Barthel index, Activity of daily living, Prognosis

## Abstract

**Background:**

This study aims to evaluate the ADL(activity of daily living) of patients with acute cerebral infarction through BI scoring, in order to observe its predictive value in the prognosis of these patients.

**Methods:**

According to the inclusion and exclusion criteria, patients with acute anterior circulation cerebral infarction were included in the present study. Then, the BI scoring was analyzed through five grades, in order to further investigate the dose-response relationship between BI scoring and mortality risk in patients with cerebral infarction. The receiver operating characteristic (ROC) curves for BI-scored patients were drawn, and the predictive authenticity of the Barthel scale in prognostic prediction for patients with cerebral infarction was estimated.

**Results:**

The difference in BI scores between the survival group and death group were statistically significant (t = 10.029, *P <* 0.05), in which the score was lower in the death group than in the survival group. According to the linear trend *×*^*2*^-test, the decrease in BI score indicates an increase in mortality risk in patients with cerebral infarction. The area under the curve (AUC) of the ROC curve was 0.794 with a *P*–value of < 0.05.

**Conclusion:**

BI scoring is a highly valuable scoring system for the prognostic prediction of patients with acute cerebral infarction.

## Background

It has been reported that cerebrovascular accidents presently rank at the top of the list of deadly or disabling diseases in adults [1]. Along with the global trend of population aging, 23 million people are estimated to suffer from cerebral strokes, with 7.8 million people dying from 2002 to 2030. Approximately 3/4 of survivors would be incapacitated in varying degrees, and the rate of severe disability will reach as high as 40% or more [2]. Post-stroke dysfunction mainly includes disorder in movement, language and cognition. This would seriously affect the patient’s activity of daily living (ADL) and social participation, and bring heavy burden to not only the patients themselves and their families, but also to the economy and healthcare system as a whole. Since the course of cerebral stroke is relatively complex, it remains as one of the most important subjects, and clinical scientists need to continuously improve its accuracy of disease evaluation and prognostic prediction in stroke patients. Over the past decade, stroke mortality has declined due to the intervention of stroke risk factors and the application of more effective treatment methods. However, this may lead to the long-term disability of those stroke patients prior to death, adding to the economic burden of individuals and society [3].

The ADL of stroke patients has been considered as one of the most basic evaluation items, and the main objective for rehabilitation. It has been chiefly used in evaluating the degree of nervous functional defects in stroke patients, the degree of dependence on others, and the functional burden induced to their families and society. However, the prognostic prediction of ADL for patients with acute cerebral infarction has been rarely reported. There are presently many different approaches for evaluating ADL. At present, the Barthel index (BI) has been widely used in the ADL evaluation of stroke patients during the phases of drug treatment [4] and rehabilitation [5]. This study aims at evaluate the ADL of patients with acute cerebral infarction through BI scoring, in order to observe its predictive value in the prognosis of these patients.

## Methods

### Study object

The present study selected patients with acute anterior circulation infarction, who were hospitalized in the Neurology Department of the Affiliated Hospital to North China University of Science and Technology from January 2012 to December 2016. The inclusion criteria were as follows: (1) patients admitted within three days after onset; (2) patients who met the standard of diagnosis for cerebral infarction formulated on the 4th National Cerebrovascular Disease Conference 1995, and confirmed by cerebral computed tomography (CT) and magnetic resonance imaging (MRI). The exclusion criteria were as follows: (1) patients with transient ischemic attacks; (2) patients with acute posterior circulation cerebral infarction; (3) patients with serious heart, liver, kidney and other organ dysfunction, or patients who cannot take care of themselves before onset; (4) patients with stroke history, and cannot take care of themselves; (5) patients who could not cooperate or could not complete the test; (6) patients who did not sign informed consent. The surviving patients after treatment were classified as the survival group, while patients who died were classified as the death group. The present study was approved by the Ethics Committee of the Affiliated Hospital of North China University of Science and Technology, Tangshan, China, and complied with the 1964 Helsinki declaration and its later amendments. A written informed consent was obtained from all patients enrolled in the present study.

### Research content

The ADL of all patients were evaluated through BI scoring [6] after admission to the hospital, including eating, bathing, making-up, dressing, bowel movement control, urinary control, toileting, moving of bed and chair, walking, walking up and down the stairs, and other 10 items. Each item was classified into four grades of 15, 10, 5 and 0, based on whether they were in need of any help, and how much help they needed. Out of 100 points, 100 points means that the patient commands a good ADL and does not need any help from others, while 0 points means that the patient is unable to live independently and needs help for all aspects of life. The BI scores were divided into five grades for the subgroup analysis: 0–20, 25–40, 45–60, 65–80, and 85–100. All assessments were conducted by a trained review team consisting of two deputy chief physicians and nurses, who were responsible for completing the evaluation of the selected cases in strict accordance with the various scales of marks.

### Statistical methods

The age and gender ratio between the survival group and death group were analyzed by chi-square test, while BI scoring was analyzed by *t*-test. The comparison of death risk ratios between groups was analyzed by linear trend *×*^*2*^-test. Furthermore, the dose-response relationship between BI scoring and death risk in patients with cerebral infarction were analyzed, a gold standard for determining the prognosis of patients based on whether the patients survived or died during their hospital stay was established, and the receiver operating characteristic (ROC) curve for BI-scored patients was drawn after admission to the hospital. Since a lower BI score indicates a worse disease, 100-BI scores were used to draw the ROC curve for BI scoring, in order to make it meet the principle, “the lower the score, the worse the disease becomes”. Then, the AUC of the curve was calculated and compared between the AUCs of varying scoring systems and the baseline area (0.5), in order to determine whether the difference was statistically significant. SAS 9.3 software was used for the analysis, and all tests were two-tailed tests with a significance level (*P*-value) of 0.05.

## Results

### General

The present study included 392 patients, which comprised of 216 male and 176 female patients. Among these patients, 276 patients were assigned to the survival group, while 116 patients were assigned to the death group, and the average age of these patients was 64.82 ± 11.40 years old. There was no significant difference in age and gender between the death group and survival group (Table 1). The BI scores in the survival group and death group were 51.92 ± 29.60 and 21.55 ± 26.37, respectively, and the difference was statistically significant (t = 10.029, *P* < 0.05), in which the score was significantly lower in the death group than in the survival group.

**Table 1:** Age and gender composition of patients with different results

Age	Survival Group (*n* = 278)		Total	Death Group (*n* = 121)		Total
	Male	Female		Male	Female	
≤60	67	44	111	17	19	36
> 60	88	77	165	44	36	80
Total	155	121	276	61	55	116

Note: Gender X^2^ = 0.050, *P* = 0.823; Age X^2^ = 4.010, *P* = 0.059

### Dose-response relationship between the BI score and mortality risk in patients with cerebral infarction

Next, the correlation intension between the BI score and mortality risk in acute cerebral infarction was further analyzed. According to the linear trend *X*^*2*^-test, the decrease in BI score indicated an increase in mortality risk in patients with cerebral infarction (Table 2).

**Table 2:** Death risks in each subgroup of patients with acute cerebral infarction through BI scoring

	BI scores of each subgroup				
	0~20	25~40	45~60	65~80	85~100
Number of the Dead	77	20	7	6	6
Number of Survivors	46	78	40	60	52

Note: χ^2^ = 96.794, *P* < 0.001

### BI scoring for comparing patient fatality rate in each subgroup of inpatients

In order to further observe the correlation intension between ADL and patient fatality rates, BI was divided into five subgroups, each with a distance of 20 points. According to the linear trend *X*^*2*^-test, the decrease in BI score indicates an increase in patient fatality rate. This proves that there is a significant dose-effect relationship between these two (Tables 3 and 4).

**Table 3:** Case fatality rates in each subgroup of inpatients with acute cerebral infarction through BI scoring

Disease Type	BI scores of each subgroup					x^2^	*P*
	0~20	25~40	45~60	65~80	85~100		
Cerebral	62.60%	20.41%	14.89%	9.10%	10.34%	96.794	< 0.001
Infarction	(77/46)	(20/78)	(7/40)	(6/60)	(6/52)		

Note: Within () are the numbers of the dead/ survival cases

**Table 4:** Hospitalization time for patients with different results

	Hospitalization time of each group(d)				
	0~7	8~15	16~21	22~30	> 30
Survival Group	101	98	57	22	0
Death Group	4	16	42	53	6

Note: χ^2^ = 136.427, *P* < 0.001

### Authenticity of the 100-BI scoring system in the prognostic prediction of cerebral infarction

**Fig. 1:**
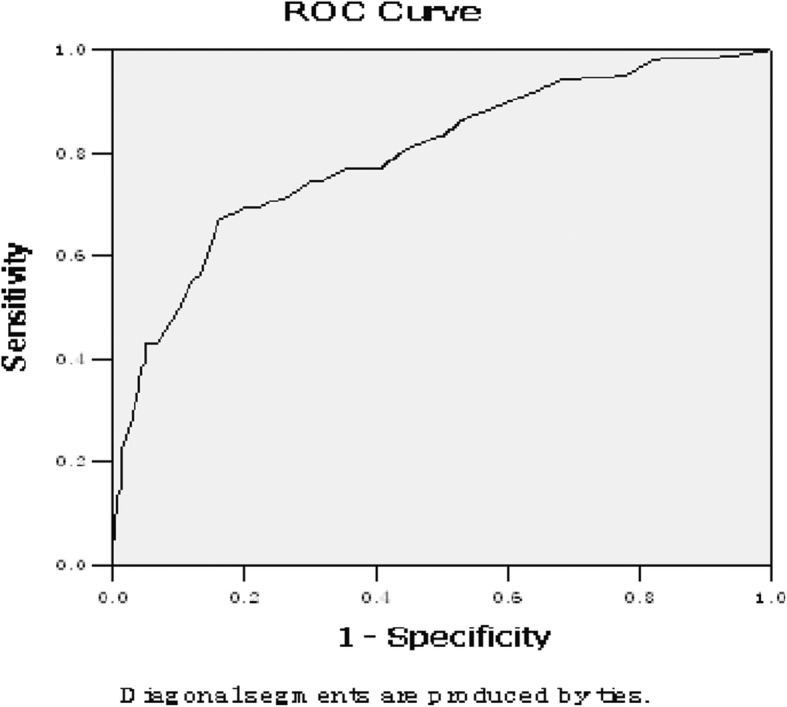
The ROC curve of ADL scoring in patients with acute cerebral infarction. ROC:receiver operating characteristic. ADL:activity of daily living. AUC: Area Under Curve. *p* < 0.05 is statistically significant

The 100-BI rating scale was assessed after admission to the hospital then ROC curve was constructed. The sensitivity of the curve was 0.782, the specificity was 0.833, the AUC was 0.794, and the *P* value was 0.02. This indicates its authenticity in prognostic prediction of acute cerebral infarction. Cerebral Infarction has a high mortality rate, and missed diagnosis is very harmful. We need to improve sensitivity and reduce misdiagnosis as much as possible. Therefore, we choose the BI score with sensitivity greater than 0.8 as a reference. Combined with clinical experience and data from this study, patients with a score below 45 are high-risk deaths. But this result also needs further research from a large multi-center sample.(Fig. 1).

## Discussion

ADL ability is essential to everyone, since it is the most basic and common activity repeatedly conducted by people in their daily life. It is not only one of the most basic indicators that reflect the living standard, but is also the main objective for post-stroke rehabilitation intervention. The BI score adopted in the present study was easy to operate, time-saving, and had features of test-retest. The ADL problems in patients can be accurately identified as soon as possible by selecting a qualified ADL scale, in order to follow correct instructions for the rehabilitation of patients, and objectively evaluate the effect of rehabilitation. Furthermore, it also helps patients with function recovery, in order to enable them to live on their own and get back to their normal life as soon as possible. In addition, it has reliability, content consistency, common validity, and a certain degree of predictive validity [7]. Therefore, it plays an important role in the present clinical research. Moreover, it has been found in a number of studies that there was no difference in the results of BI scoring, when compared to those of other ADL evaluation indicators [8–10].

According to the present study, it was found that the BI score in the death group was significantly lower than that in the survival group. Furthermore, the patient fatality rates of inpatients decreased with the increase in BI score, indicating that there was a significant dose-effect relationship between these two. The AUC of the ROC curve of the 100-BI scoring was 0.794. This shows that it has a predictive value in the prognostic evaluation of patients with acute cerebral infarction.

The results of the present study are consistent with those of previous studies. According to a study on 75 stroke patients conducted by Martinsson et al. [11], baseline BI scores were higher in survivors after one week and three months, when compared to those in the death group. Furthermore, Rollnik et al. found that patients with a BI score of ≥40 were more likely to be discharged from the hospital, and patients with a BI score of ≥60 had shorter hospital stays [12]. A survey on post-discharge rehabilitation in stroke patients revealed that patients with a BI score of ≥60 before discharged maintained their scores at a relatively high level six months later, and patients with a BI score of ≤40 also demonstrated no significant changes in their scores, which remained on a relatively low level six months later [13–16]. Wade’s follow-up study revealed that the lower the baseline BI score was, the higher the death rate was for stroke patients six months later, when combined with worsening body function [17]. The ROC curve has been widely used in evaluating medical diagnostic performance [18]. The present study found that the AUC of the ROC curve for the 100-BI score was 0.794, which was consistent with the findings of Mar et al. Furthermore, the follow-up study revealed that the AUC of the ROC curve of BI scoring for predicting the mortality rate after one year was 0.835 [19–23].

At present, there are few studies on the prediction accuracy of BI scoring for predicting the recent death of patients with acute cerebral infarction. The present study found that BI scoring was highly valuable as a scoring system in prognostic prediction on patients with acute cerebral infarction. Due to the fact that the scale evaluation was largely influenced by subjective factors, sometimes the prognostic evaluation cannot reply solely on the detailed scoring of the nervous system in clinical practice. Hence, other situations should be taken into account. In addition, the examinees selected for the present study were patients with an onset within three days. Therefore, bias might exist in terms of comparison. Taking into account the fact that the condition of patients with cerebral infarction during the acute phase was unstable, most studies are inclined to the view that the evaluation on patients should last for several days, or even longer [4]. Therefore, prospective studies with larger sample sizes are needed to verify this.

## Conclusion

BI scoring is a highly valuable scoring system in prognostic prediction for patients with acute cerebral infarction.

## Data Availability

The datasets used and/or analysed during the current study available from the corresponding author on reasonable request.

## Abbreviations

ADL: Activity of daily living
BI: Barthel index
ROC: Receiver operating characteristic

## References

[1] Mendis S (2013). Stroke disability and rehabilitation of stroke: World Health Organization perspective [J]. Int J Stroke.

[2] Mathers CD, Loncar D (2006). Projections of global mortality and burden of disease from 2002 to 2030[J]. PLoS Med.

[3] Di Carlo A, Lamassa M, Pracucci G, Basile AM, Trefoloni G, Vanni P (1999). Stroke in the very old : clinical presentation and determinants of 3-month functional outcome: a European perspective. European BIOMED study of stroke care group [J]. Stroke.

[4] Duncan PW, Lai SM, Keighley J (2000). Defining post-stroke recovery: implications for design and interpretation of drug trials [J]. Neuropharmacol.

[5] Roberts L, Counsell C (1998). Assessment of clinical outcomes in acute stroke trials [J]. Stroke.

[6] Zhang XY, Shen JY, Zhang S (2017). Effect of clopidogrel combined with atorvastatin on NIHSS and Barthel score in patients with progressive cerebral infarction [J]. J Hainan Med Univ.

[7] Wade DT, Collin C (1988). The Barthel ADL index: a standard measure of physical disability?[J]. Int Disabil Stud.

[8] Gosman-Hedstrom G, Svensson E (2000). Parallel reliability of the functional independence measure and the Barthel ADL index [J]. Disabil Rehabil.

[9] Houlden H, Edwards M, Mcneil J, Greenwood R (2006). Use of the Barthel index and the functional Independence measure during early inpatient rehabilitation after single incident brain injury [J]. Clin Rehabil.

[10] Mahoney FI, Barthel DW (1965). Fuction evaluation: the barthel index [J]. Md State Med J.

[11] Martinsson L, Eksborg S (2006). Activity index - a complementary ADL scale to the Barthel index in the acute stage in patients with severe stroke [J]. Cerebrovasc Dis.

[12] Rollnik JD (2009). [Barthel index as a length of stay predictor in neurological rehabilitation][J]. Rehabilitation (Stuttg).

[13] Nakao S, Takata S, Uemura H, Kashihara M, Osawa T, Komatsu K (2010). Relationship between Barthel index scores during the acute phase of rehabilitation and subsequent ADL in stroke patients [J]. J Med Investig.

[14] Della Pietra GL, Savio K, Oddone E, Reggiani M, Monaco F, Leone MA (2011). Validity and reliability of the Barthel index administered by telephone [J]. Stroke.

[15] Quinn TJ, Langhorne P, Stott DJ (2011). Barthel index for stroke trials:development, properties, and application [J]. Stroke.

[16] Huybrechts KF, Caro JJ (2007). The Barthel index and modified Rankin scale as prognostic tools for longterm outcomes after stroke: a qualitative review of the literature [J]. Curr Med Res Opin.

[17] Wade DT, Hewer RL (1987). Functional abilities after stroke: measurement, natural history and prognosis [J]. J Neurol Neurosurg Psychiatry.

[18] Metz CE (1989). Some practical issues of experimental design and data analysis in radiological ROC studies [J]. Investig Radiol.

[19] Mar J, Masjuan J, Oliva-Moreno J, Gonzalez-Rojas N, Becerra V, Casado MÁ (2015). CONOCES Investigators Group. Outcomes measured by mortality rates, quality of life and degree of autonomy in the first year in stroke units in Spain [J]. Health Qual Life Outcomes.

[20] Quinn TJ, Dawson J, Walters MR, Lees KR (2009). Functional outcome measures in contemporary stroke trials [J]. Int J. Stroke.

[21] De Wit L, Putman K, Devos H, Brinkmann N, Dejaeger E, De Weerdt W (2014). Long-term prediction of functional outcome after stroke using single items of the Barthel Index at discharge from rehabilitation centre [J]. Disabil Rehabil.

[22] Kwakkel G, Veerbeek JM, Harmeling-van der Wel BC, van Wegen E, Kollen BJ (2011). Early Prediction of functional Outcome after Stroke (EPOS) Investigators. Diagnostic accuracy of the Barthel Index for measuring activities of daily living outcome after ischemic hemispheric stroke:does early poststroke timing of assessment matter?[J]. Stroke.

[23] Cai YF, Jia Z, Li WF, Wen LL, Zhang YT, Guo JW (2007). Multicenter evaluation of the ischemic stroked patients with the Chinese Barthel index: a prognostic study [J]. Chin J Cerebrovasc Dis.

